# Alveolar nitric oxide and its role in pediatric asthma control assessment

**DOI:** 10.1186/1471-2466-14-126

**Published:** 2014-08-04

**Authors:** Olaia Sardón, Paula Corcuera, Ane Aldasoro, Javier Korta, Javier Mintegui, José I Emparanza, Eduardo G Pérez-Yarza

**Affiliations:** 1 Division of Pediatric Respiratory Medicine, Hospital Universitario Donostia, Avda. Dr. Beguiristain número 118, San Sebastian, Guipúzcoa CP 20014, Spain; 2 Department of Pediatrics, University of the Basque Country (UPV/EHU), San Sebastian, Spain; 3 Epidemiology Unit (CIBER-ESP), Donostia University Hospital, San Sebastian, Spain; 4 Biomedical Research Centre Network for Respiratory Diseases (CIBERES), San Sebastián, Spain

## Abstract

**Background:**

Nitric oxide can be measured at multiple flow rates to determine proximal (maximum airway nitric oxide flux; Jaw_NO_) and distal inflammation (alveolar nitric oxide concentration; CA_NO_). The main aim was to study the association among symptoms, lung function, proximal (maximum airway nitric oxide flux) and distal (alveolar nitric oxide concentration) airway inflammation in asthmatic children treated and not treated with inhaled glucocorticoids.

**Methods:**

A cross-sectional study with prospective data collection was carried out in a consecutive sample of girls and boys aged between 6 and 16 years with a medical diagnosis of asthma. Maximum airway nitric oxide flux and alveolar nitric oxide concentration were calculated according to the two-compartment model. In asthmatic patients, the asthma control questionnaire (CAN) was completed and forced spirometry was performed. In controls, differences between the sexes in alveolar nitric oxide concentration and maximum airway nitric oxide flux and their correlation with height were studied. The correlation among the fraction of exhaled NO at 50 ml/s (FE_NO50_), CA_NO_, Jaw_NO_, forced expiratory volume in 1 second (FEV_1_) and the CAN questionnaire was measured and the degree of agreement regarding asthma control assessment was studied using Cohen’s kappa.

**Results:**

We studied 162 children; 49 healthy (group 1), 23 asthmatic participants without treatment (group 2) and 80 asthmatic patients treated with inhaled corticosteroids (group 3). CA_NO_ (ppb) was 2.2 (0.1-4.5), 3 (0.2-9.2) and 2.45 (0.1-24), respectively. Jaw_NO_ (pl/s) was 516 (98.3-1470), 2356.67 (120–6110) and 1426 (156–11805), respectively. There was a strong association (r = 0.97) between FE_NO50_ and Jaw_NO_ and the degree of agreement was very good in group 2 and was good in group 3. There was no agreement or only slight agreement between the measures used to monitor asthma control (FEV_1_, CAN questionnaire, CA_NO_ and Jaw_NO_).

**Conclusions:**

The results for CA_NO_ and Jaw_NO_ in controls were similar to those found in other reports. There was no agreement or only slight agreement among the three measure instruments analyzed to assess asthma control. In our sample, no additional information was provided by CA_NO_ and Jaw_NO_.

## Background

Asthma is a chronic inflammatory disease characterised by recurrent symptoms of cough, wheezing and/or respiratory distress, associated with variable airway obstruction and bronchial hyperresponsiveness [1].

The Global Initiative for Asthma (GINA) [2] indicates that the severity of asthma should be determined on the basis of the degree of control in the corresponding treatment step, which is achieved by assessing the frequency of symptoms, the need for rescue bronchodilators, and pulmonary function [3]. To assess deterioration, questionnaires can be used that evaluate patients’ perceptions of their disease control. The only questionnaire developed and validated in the Spanish pediatric population is the CAN questionnaire [4].

Our group recently studied the association among symptoms, pulmonary function, and fraction of exhaled nitric oxide (FE_NO_) for the management of asthma in children [5] and, like other authors [6], we found that the association–despite being significant–was weak.

The growing interest in modelling exhaled nitric oxide is understandable because only an extended NO analysis can reveal where in the lung the NO production is altered. FE_NO_ is inherently non-specific regarding the origin of NO in the lungs and the recommended exhalation flow of 50 ml/s (FE_NO,50_) identifies inflammatory activity mainly in the proximal airway or bronchi but the distal contributions are effectively ignored. However, applying mathematical models, the NO signal con be partitioned into proximal [maximum airway NO flux (J’aw_NO_)] and distal (CA_NO_) airway which could indirectly reflect inflammatory activity in more distal areas (alveolar-capillary interface) [7]. There is a lack of standardization in the technique to determine CA_NO_ and J’aw_NO._ In this study, the two-compartment model and Tsoukias and George’s equation was applied [8], however, other, more complex models have been developed [9-11]. Several authors have reported an association between elevated CA_NO_ values and poor asthma control [12,13], persistent nocturnal symptoms, severe treatment-refractory asthma, and the risk of exacerbations [14].

The main aim of the present study was to study the association and correlation among symptoms, pulmonary function, proximal [maximum airway NO flux (J’aw_NO_)] and distal (CA_NO_) airway inflammation with a view to aiding the management of asthma in daily clinical practice. The second objective was to determine alveolar NO in a healthy population.

## Methods

### Design of the study

A cross-sectional study with prospective data collection was carried out in a consecutive sample of girls and boys aged between 6 and 16 years with a medical diagnosis of asthma according to GINA 2012 criteria [2], recruited in the outpatient clinic of the Pediatric Pneumology Section of Donostia Hospital between January and August 2012.

### Study population

Untreated asthmatic patients and asthmatic patients receiving inhaled glucocorticoid therapy as part of their standard care were included. Patients were excluded if they had asthma exacerbations or acute respiratory infection at the consultation. A control group was consecutively recruited during the same time period consisting of healthy girls and boys aged between 6 and 16 years. In this group, care was taken to ensure the absence of asthma, allergic rhinitis, food allergy or atopic dermatitis in the clinical history and on physical examination.

Primary exclusion criteria consisted of patients not meeting the inclusion criteria, those with associated diseases, those who were incapable of collaborating and/or children, parents and/or guardians who refused to participate.

### Definitions

Asthma severity and control were classified according to the GINA 2012 criteria [2]. Among the asthmatic group, allergic rhinitis was considered to be present when there were signs and symptoms compatible with this diagnosis, a positive result to one or more aeroallergens in serum-specific IgE testing (class III or higher) and/or a positive skin prick test; food allergy when there were signs and symptoms compatible with specific IgE in blood (class III or higher), and atopic dermatitis when there were compatible signs and symptoms [2,3].

### Methodology

In both groups, CA_NO_ and J’aw_NO_ were determined through the multiple exhalation flow technique at 50, 100 and 200 ml/s. Measurements were made by on-line recording and the stationary chemiluminescence analyzer, Eco Medics CLD 88 SP®, was used with DENOX 88 adaptive flow control. Flow and volume were calibrated daily and NO gas was also calibrated monthly.

All children were instructed to exhale, starting from the level of maximum inspiration, at 3 constant expiratory flow rates (50, 100 and 200 ml/s). The manoeuver was performed in triplicate, with calculation of the mean value of the three measurements obtained for each flow rate. First, determinations were performed at a flow rate of 50 ml/s, followed by 100 ml/s and finally at 200 ml/s. All measurements were made in accordance with the recommendations of the European Respiratory Society (ERS) and the American Thoracic Society (ATS), published in 2005 [15]. The coefficient of variability among the three determinations had to be within 10%. After NO determinations had been obtained at different flow rates, CA_NO_ and J’aw_NO_ were calculated by applying the two-compartment model and Tsoukias and George’s equation [8].

In all asthmatic patients, after NO determination, forced spirometry was performed (MasterLab. Version 5.3. Viasis**®**, Wuerzburg, Germany) according to ATS/ERS recommendations [16]. The equations proposed by Zapletal were used to calculate the percentage of normality [17,18].

A medical history and physical examination were also performed and the asthma control questionnaire (CAN) [4] was completed by the parents (children younger than 9 years) or by the children and adolescents (older than 9 years). The CAN questionnaire consists of nine questions that explore various aspects of asthma control in the previous 4 weeks. Responses are coded numerically and a total score is calculated, ranging from 0 (better control) to 36 (worse control). The questionnaires were delivered and collected before the clinical evaluation and lung function tests.

### Statistical analysis

The quantitative variables analysed were age (years), weight (kg), height (cm), NO determination (ppb) at 3 expiratory flow rates (FE_NO,50_, FE_NO,100_, FE_NO,200_), CA_NO_ (ppb) and J’aw_NO_ (pl/s). In the asthmatic group, the following variables were also gathered: CAN questionnaire score (points), forced expiratory volume in 1 second (FEV_1_ as a percentage of the predicted value), forced vital capacity (FVC as a percentage of the predicted value), the FEV_1_/FVC ratio (as a percentage of the predicted value) and forced expiratory flow between 25% and 75% of FVC (FEF_25–75_ as a percentage of the predicted value).

The qualitative variables studied were sex and personal atopy (atopic dermatitis, allergic rhinitis, food allergy and aeroallergen sensitization). In asthmatic participants, asthma severity was also analysed, as well as inhaled corticosteroid therapy and the degree of control.

Spearman’s rho was used to analyse the association between CA_NO_ and J’aw_NO_ with FE_NO.50,100,200_, FEV_1_ and the CAN questionnaire. Given that personal atopy and current treatment with inhaled glucocorticoids can act as confounding factors in the FE_NO,50_, CA_NO_ and J’aw_NO_ values obtained, the statistical analysis was adjusted by these variables using multiple lineal regression.

Cohen’s kappa coefficient was used to assess the degree of agreement, with categorization of the variables according to normal values, between CA_NO,_ J’aw_NO,_ FEV_1_, FE_NO,50_, and the CAN questionnaire.

In line with prior publications, the cutoff point for normal values was defined as ≤ 25 ppb for FE_NO,50_[19,20], ≥80% for the relative value of FEV_1_ (% predicted) [1,2] and a score of less than 8 points for the CAN questionnaire [4]. For CA_NO_ and J’aw_NO_, the cut-off for normal values was established on the basis of the upper limit of the control group (<4.5 ppb for CA_NO_ and <1470 pl/s for J’aw_NO_).

In the control group, the Mann–Whitney test was used to study differences in CA_NO_ and J’aw_NO_ by sex and Spearman’s rho was used to study the association between height and CA_NO_ and between height and J’aw_NO_.

Chi-square test was used for qualitative variables (sex), Student’s t-test and ANOVA were used for quantitative variables which followed normal distribution (age, weight, and height) and Kruskal Wallis and Mann–Whitney U test were used for quantitative variables which did not follow normal distribution (FE_NO,50_ J’aw_NO_ CA_NO_).

### Sample size

The sample size was estimated based on the correlation coefficients expected according to published data [12-14,21]. An alpha level of 5% was established for all tests and the SYSTAT 9.0™ was used for the statistical analysis.

### Ethics

This study was approved by the Ethics and Research Committee at the Donostia University Hospital. Informed consent was obtained from all participants. The parents’ and/or guardians’ permission, as well as that of the participating child, if required by current legislation, was obtained for data exploitation.

## Results

### Characteristics of the study population

The cohort consisted of 162 participants. In 158 (97.5%), all determinations were successfully completed, distributed in group 1 (healthy controls, n = 49 [32.2%]), group 2 (untreated asthmatic patients, n = 23 [15.1%]) and group 3 (asthmatic patients receiving inhaled glucocorticoid therapy, n = 80 [52.5%]).

Four participants (2.4%) were excluded due to poor technique and a further 6 participants (100% asthmatic) were excluded because the NO determination did not follow the linear model (negative CA_NO_ values). Age, weight, FE_NO,50_ and spirometry were analysed in these individuals and no significant differences were found compared with included participants. The characteristics of the study population are shown in Table 1.

**Table 1:** Characteristics of the study population Group 1 (healthy controls), group 2 (untreated asthmatic patients) and group 3 (asthmatic patients receiving inhaled glucocorticoid therapy)

	**Group 1 (N = 49)**	**Group 2 (N = 23)**	**Group 3 (N = 80)**	**p**
**Age** (years) (mean ± SD)	10.1 ± 1.9	9.3 ± 2.06	10.7 ± 2.85	NS
**Sex** F/M	28/21	10/13	29/51	NS
**Weight** (kg) (mean ± SD)	38 ± 13.9	36 ± 10.3	42.1 ± 15.2	NS
**Height** (cm) (mean ± SD)	139.4 ± 13.2	137.7 ± 11.5	144.84 ± 16.2	NS
**Atopic dermatitis** N (%)	0	8 (34.7)	30 (37.5)	
**Allergic rhinitis** N (%)	0	19 (82.6)	70 (87.5)	
**Food allergy** N (%)	0	0	12 (15)	
**Mild asthma** N (%)	0	19 (82.6)	57 (71.2)	
**Moderate asthma** N (%)	0	4 (17.4)	23 (28.7)	NS
**Degree of control** N (%)				
- Good	0	15 (65.2)	63 (78.7)	NS
- Partial	0	6 (26)	11 (13.7)	
- Poor	0	2 (8.7)	6 (7.5)	
**ICS dose** (fluticasone mcg) (mean ± SD)	0	0	135.45 (35.2)	
**CAN** (median and range)	0	5 (0–29)	5 (0–27)	NS
**FEV**_**1**_ (mean ± SD)	0	98.17 (15.07)	99.65 (10.59)	NS
**FE**_**NO,50**_ (ppb) (median and range)	11.5 (1.6-27.3)	48.3 (7.4-122)	32 (3.5-234)	p = 0.002 (2 vs 3) p < 0.001 (1 vs 2 and 3)
**J’aw**_**NO**_ (pl/s) (median and range)	516 (98.3-1470)	2356.7 (120–6110)	1426 (156–11805)	p = 0.001 (2 vs 3) p < 0.001 (1 vs 2 and 3)
**CA**_**NO**_ (ppb) (median and range)	2.2 (0.1-4.5)	3.0 (0.2-9.2)	2.4 (0.1-24)	NS ( 2 vs 3) P = 0.022 (1 vs 2 and 3)

F: female; M: male; SD: standard deviation; J’aw_NO_: maximum airway NO flux; CA_NO_: alveolar nitric oxide concentration; FE_NO,50_: fraction of exhaled nitric oxide at a flow rate of 50 ml/s; CAN: asthma control questionnaire; FEV_1_: forced expiratory volume in 1 second; ppb: parts per billion; pl/s: picolitre/second; NS: no significant differences (p > 0,05); vs: versus.

No significant differences were found in age, weight, height or sex among the 3 study groups. In addition, no differences were found in gender and height between asthma (group 2 y 3) and control group (group 1) (p = 0.21 and p = 0.15 respectively). Moreover, there were no significant differences in gender and height between untreated asthmatic patients (group 2) and asthmatic patients receiving inhaled glucocorticoid therapy (group 3) (p = 0.31 and p = 0.19 respectively).

In the control group, no significant differences were found in CA_NO_ or J’aw_NO_ by sex.

Similarly, no statistically significant associations were found between height and J’aw_NO_ (r = 0.15, p > 0.05) or between height and CA_NO_ (r = 0.22, p > 0.05).

### Asthmatic patients

In general, asthmatic participants had mild asthma that was well controlled [median CAN questionnaire score: 5 (0–29)] and normal baseline spirometry (mean FEV_1_ = 99.7%; mean FEV_1_/FVC = 85%). Asthmatic participants receiving no treatment of any type (group 2) had higher CA_NO_, J’aw_NO_ and FE_NO,50_ values than asthmatic participants receiving inhaled glucocorticoid therapy (group 3): CA_NO_ (median and range) 3 ppb (0.2-9.2), J’aw_NO_ 2356.67 pl/s (120–6110) and FE_NO,50_ 48.3 ppb (7.4-122) versus CA_NO_ 2.4 ppb (0.1-24), J’aw_NO_ 1426 pl/s (156–11805) and FE_NO,50_ 32 ppb (3.5-234). This difference was statistically significant for J’awNO (p = 0.001) and FE_NO,50_ (p = 0.002) (Table 1).

**Figure 1:**
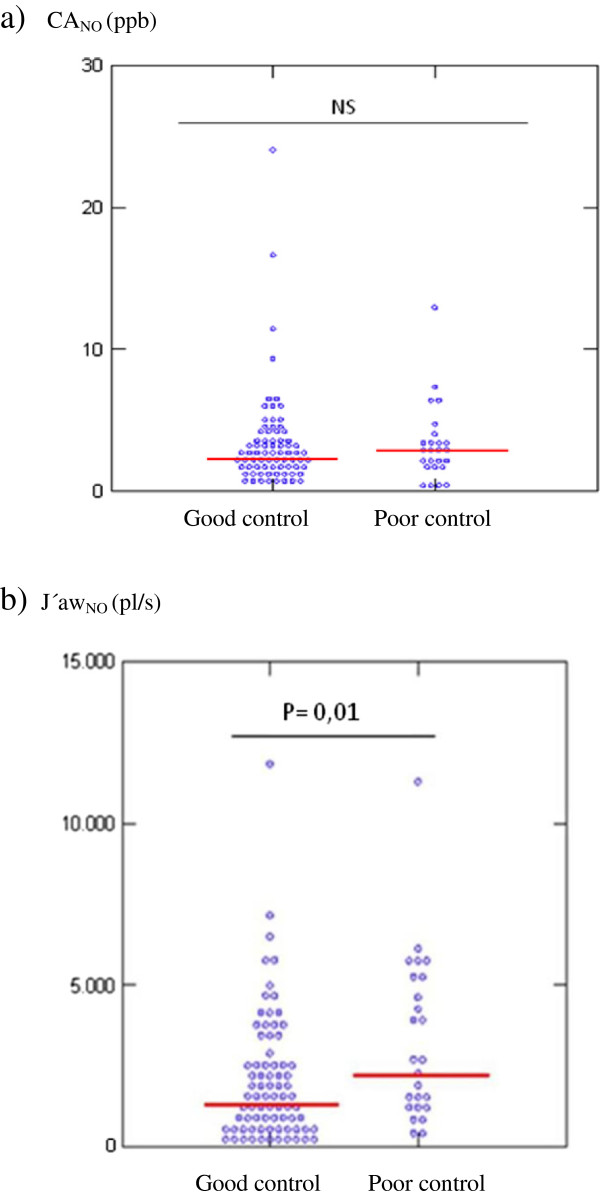
**CA**_**NO **_**and J’aw**_**NO **_**in asthmatic patients with good asthma control versus those with poor or partial control. a)**. CA_NO_ (ppb), Good control Poor control. **b)**. J’aw_NO_ (pl/s), Good control Poor control. CA_NO_: alveolar nitric oxide concentration; ppb: parts per billion; J’aw_NO_: maximum airway NO flux; pl/s: picolitres/second; NS: non significant.

Asthmatic participants with poor or partial asthma control [2] (n = 25; 24.2%) had higher CA_NO_, J’aw_NO_ and FE_NO,50_ values than asthmatic participants with good control [2] (n = 78; 75.7%): CA_NO_ (median and range) 3.1 ppb (0.1-16.6), J’aw_NO_ 2576 pl/s (413–11263) and FE_NO,50_ 51.9 ppb (8.6-209) versus CA_NO_ 2.3 ppb (0.1-24), J’aw_NO_ 1445 pl/s (120–11805) and FE_NO,50_ 32.5 ppb (3.5-234). This difference was not statistically significant (p = 0.4) for CA_NO_, but was statistically significant for J’aw_NO_ (p = 0.01) and FE_NO,50_ (p = 0.006) (Figure 1). No significant differences (p > 0.05) were found for any of the spirometric variables between the group of patients with good asthma control and the group with poor or partial asthma control.

### Differences between asthmatic patients and healthy participants

FE_NO,50_ was significantly higher in the asthmatic groups (groups 2 and 3) than in the group of healthy participants (group 1): median and range; 35.4 ppb (3.5-234) versus 11.5 ppb (1.6-27.3) (p < 0.001). J’aw_NO_ was also higher in the asthmatic group than in the control group; 1703 pl/s (120–11805) versus 516 pl/s (98.33-1470) (p < 0.001). Similarly, CA_NO_ was higher in the asthmatic group than in the control group: 2.7 ppb (0.1-24) versus 2.2 ppb (0.1-4.5) (p = 0.022) (Table 1). We did also a separate analysis between group 1 (healthy participants) and group 2 (untreated asthmatic patients) and no significant differences were found among the results obtained.

### Association and degree of agreement among FE_NO,50_, CAN, FEV_1_, CA_NO_ and J’aw_NO_

A close and significant association was found between J’aw_NO_ and FE_NO,50_ (r = 0.97; p < 0.05). No association (CA_NO_ and J’aw_NO_), or only a weak association, was found between J’aw_NO_ and CAN scores, J’aw_NO_ and FEV_1_, CA_NO_ and FEV_1_, CA_NO_ and CAN scores and between FEV_1_ and CAN scores. On categorizing the variables (FE_NO,50_, CAN questionnaire, FEV_1,_ CA_NO_ and J’aw_NO_) according to normal values, there was an optimal degree of agreement between FE_NO,50_ and J’aw_NO_, in the 2 groups of asthmatic patients. This agreement was almost perfect for group 2 (KC = 0.89) and was substantial (KC = 0.71) for group 3. No agreement, or only slight agreement to establish the degree of asthma control was found between J’aw_NO_ and CAN scores (KC = 0.34), J’aw_NO_ and FEV_1_ (KC = 0.123), CA_NO_ and FEV_1_ (KC = 0.104), CA_NO_ and CAN scores (KC = 0.03), CA_NO_ and J’aw_NO_ (KC = 0.074) and between FEV_1_ and CAN scores (KC = 0.12).

The statistical analysis was adjusted by current treatment with inhaled glucocorticoids using multiple lineal regression and no significant differences were found among the results obtained.

## Discussion

Several guidelines and international consensus documents for the management of asthma recommend evaluation of clinical symptoms and lung function to establish the degree of asthma control [1,2], without including assessment of markers of inflammation such as NO, although these documents suggest the possibility of performing further studies to evaluate whether monitoring of such markers could improve asthma management in clinical practice [22]. In addition, several studies have shown that distinguishing between NO from the proximal airway (FE_NO,50_ and J’aw_NO_) and/or that from the distal airway (CA_NO,_) could be useful as a surrogate marker of airway inflammation in the assessment of asthmatic patients, although the role of this compartmentalization remains to be determined in clinical practice and there is, as yet, no standardised technique for the determination of these parameters [12,23].

In this context, the main objective of this study was to examine the association and degree of agreement among symptoms, lung function (FEV_1_), proximal (J’aw_NO_) and distal (CA_NO_) airway inflammation and asthma management in a cohort of asthmatic children and teenagers. In our sample, no additional information was provided to assess asthma control by CA_NO_ and J’aw_NO._

Like other reports [3,21,24], in our cohort of asthmatic patients (generally with mild and/or moderate asthma and mostly well controlled), there was no significant association between NO from the proximal (J’aw_NO_) and distal (CA_NO_) (r = −0.001) airway, indicating that these two determinations provide independent information. However, J’aw_NO_ and FE_NO,50_ were strongly associated and showed optimal agreement, indicating that a flow rate of 50 ml/s is sufficient for NO determination in the proximal airway. J’aw_NO_ seems to provide no additional information and consequently, FE_NO,50_ could be sufficient to characterize inflammation in the proximal airway [24]. Proximal inflammation (FE_NO,50_ and J’aw_NO_) has not consistently been associated with the degree of asthma control or with the risk of exacerbations in pediatric patients [25,26]. A possible explanation for these findings is the presence of confounding factors such as inhaled glucocorticoid therapy or atopy, which influence NO from the proximal airway. In our sample, asthmatic patients who were treated with inhaled glucocorticoids as part of their standard care received low or mild doses between 100 and 200 mcg per day. The statistical analysis was adjusted by current treatment with inhaled glucocorticoids using multiple lineal regression and no significant differences were found among the results obtained.

In agreement with the findings of other authors [12,27], in our cohort, we found no association (CA_NO_ and J’aw_NO_) or significant but weak association between J’aw_NO_ and CAN scores, J’aw_NO_ and FEV_1_, CA_NO_ and FEV_1_, CA_NO_ and CAN scores and between FEV_1_ and CAN scores probably because the measurement instruments used quantify distinct variables that influence asthma differently and at distinct times. However, other authors [12,13,21] have found differences between asthmatic patients with elevated CA_NO_ and a poor score on the Asthma Control Test (ACT) questionnaire and elevated CA_NO_ and lower FEV_1_. A possible explanation could lie in differences in the populations selected for study. Our cohort of asthmatic (treated and untreated) patients was, in general, a group with mild, well-controlled asthma [median CAN questionnaire score: 5 (0–29)] and normal baseline spirometry (mean FEV_1_ = 99.7%; mean FEV_1_/FVC = 85%). They were included consecutively without taking into account the severity of the disease. The inclusion of a population with more severe asthma could possibly have modified our results although we can not be sure. Moreover, the inclusion of more untreated asthmatic patients could also varied our results. However, no significant difference were found in age, sex, height, weight, lung function, symptoms, severity and control of asthma between treated and untreated children.

In this sense, some authors [27,28] did not found association between FE_NO,50_, CA_NO,_ the level of asthma control and severity of the disease in stable and unobstructed asthmatic children and adults. Other authors, found abnormal FE_NO,50_ but normal CA_NO_ during asthma exacerbations. Finally, Mahut et al. [29] concluded that the usefulness of alveolar nitric oxide in asthma remain to be established.

Given that some authors have shown significant differences in CA_NO_ determinations according to the method used, the second objective of the study was to obtain alveolar NO values (CA_NO_) in our healthy population with the method described previously [8]. The exclusion criteria in this group were strict, leading to a small sample (n = 49) due to the obvious limitations in this group. In our sample, values of CA_NO_ (median 2.2 ppb; range 0.1-4.5) and J’aw_NO_ (median 516 pl/s; range 98.33-1470 pl/s) were similar to those described by other authors [12,21,24,30]. In contrast, Mahut et al. [7] found higher CA_NO_ values (mean 4.2 ± 2 ppb) and lower J’aw_NO_ values (mean 320 ± 130 pl/s). These differences could be explained by the different populations studied and/or by differences in the methodology used [31].

Unlike previous studies [24,30], in our cohort of healthy children there were no differences in CA_NO_ or J’aw_NO_ in relation to height or weight.

Our results show that the two-compartmental model of NO exchange cannot be applied in approximately 6% of asthmatic patients, which, according to the literature, could be explained by differences in ventilation and inflammatory patterns in some of these patients [32]. Our percentage is somewhat lower than that reported by other authors [12,21].

One of the main limitations of this study is the lack of standardization in the technique to determine CA_NO_ and J’aw_NO._ Although it is true that two-compartment models [8] provide additional information on the degree of alveolar (distal airway) and/or bronchial (proximal airway) participation in inflammation, the simplification leads to some limitations, such as the demonstrated axial diffusion of NO and the geometry of the airways themselves, since it has been shown that there is some exchange between the two compartments and that a proportion of CA_NO_ may correspond to NO produced in the bronchial compartment which, through retrograde axial diffusion, reaches the alveoli.

Other, more complex models have been developed [9,10], as well as a model of exhalation at multiple flows that incorporates the retrograde axial diffusion model and is adapted to the trumpet shape of the airway tree [11]. Importantly, approximation of CA_NO_ and J’aw_NO_ could be influenced by the range of flows selected [8]. The inclusion of flows that are too low could overestimate CA_NO_ and underestimate J’aw_NO_ and could also be uncomfortable for pediatric patients [24]. Moreover, flow rates of around 200 ml/s is not always sufficient to achieve a stable plateau in NO concentration curve. For all these reasons, we chose to use expiratory flow rates of between 50 and 200 ml/s.

Another limitation of this study is its cross-sectional design, since we analysed a disease that varies over time by determining pulmonary function and NO concentration at a specific moment**.** Serial determinations could offer a more faithful profile of inflammation and disease control in individual patients according to their baseline values and those during exacerbations.

## Conclusions

In summary, normal values of both CA_NO_ and J’aw_NO_ obtained in this study were similar to those of other published series. There was no agreement or only slight agreement between the measures used to monitor asthma control: FEV_1_, the CAN questionnaire, CA_NO_ and J’aw_NO_. This weak agreement was probably found because these measures quantify variables that influence asthma in a different way and at distinct moments. This is a cross-sectional study and the status of the disease that varies over time was analyzed in a particular moment. Therefore, in that moment, the variables analyzed in each patient may not be concordant. Although they are complementary, none of them can be exchanged for another in the management of the disease in clinical practice. In our sample, no additional information was provided to assess asthma control by CA_NO_ and J’aw_NO_.

## Abbreviations

GINA: Global initiative for asthma; CA_NO_: Alveolar nitric oxide concentration; J’aw_NO_: Maximum airway nitric oxide flux; FEV_1_: Forced expiratory volume in 1 second; CAN questionnaire: The asthma control questionnaire; NO: Nitric oxide; FE_NO,50_: Fraction of exhaled nitric oxide at 50 ml/s; ACT: Asthma control test; CK: Cohen’s kappa coefficient.

## Competing interests

The authors declare that they have no competing interests.

## Authors’ contributions

OS participated in the design of the study and in data collection. Moreover, OS drafted the manuscript. PC participated in data collection and in the interpretation of data. AA participated in data collection. JK conceived the study and participated in its design and coordination. JM participated in data collection. JIE performed the statistical analysis. EGPY conceived the study and participated in its design and coordination. EGPY served as the guarantor of the paper and takes responsibility for the integrity of the work. All authors read and approved the final manuscript.

## Pre-publication history

The pre-publication history for this paper can be accessed here:

http://www.biomedcentral.com/1471-2466/14/126/prepub
